# The ECG Characteristics of Patients With Isolated Hypomagnesemia

**DOI:** 10.3389/fphys.2020.617374

**Published:** 2021-01-27

**Authors:** Yiheng Yang, Cheng Chen, Penghong Duan, Suman Thapaliya, Lianjun Gao, Yingxue Dong, Xiaomeng Yin, Xiaolei Yang, Rongfeng Zhang, Ruopeng Tan, Simei Hui, Yue Wang, Richard Sutton, Yunlong Xia

**Affiliations:** ^1^Department of Cardiology, First Affiliated Hospital of Dalian Medical University, Dalian, China; ^2^Department of Cardiology, First Affiliated Hospital of Xinxiang Medical University, Xinxiang, China; ^3^Department of Cardiology, Hammersmith Hospital Campus of Imperial College, London, United Kingdom

**Keywords:** electrocardiogram, serum magnesium, ventricular arrhythmia, sudden cardiac death, repolarization dispersion

## Abstract

**Background:**

Electrocardiographic (ECG) characteristics of patients with isolated hypomagnesemia are not well defined. We aimed to investigate these ECG characteristics in order to define clearly the features of isolated hypomagnesemia.

**Hypothesis:**

Lower serum magnesium could affect ECG parameters after excluding potential confounders.

**Methods:**

This retrospective study was of patients with low serum magnesium <0.65 mmol/L compared with the same patients after restoration to normal serum magnesium. Patients with hypokalemia, hypocalcemia and other electrolyte disturbances were excluded. ECG parameters manually determined and analyzed were P wave dispersion, PR interval, QRS duration, ST-T changes, T wave amplitude, T peak-to-end interval (Tpe), corrected Tpe (Tpec), QT, corrected QT (QTc), QT peak corrected (QTpc) and Tpe dispersion, Tpe/QT ratio.

**Results:**

Two-hundred-and-fourteen patients with isolated hypomagnesemia were identified with 50 of them (56.9 ± 13.6 years; 25 males) being eligible for final analysis from 270,997 patients presenting April 2011–October 2017. In the period of isolated hypomagnesemia, P wave duration was found prolonged (*p* ≤ 0.02); as was QTc (439 ± 27 vs. 433 ± 22, p = 0.01). Tpec (122 ± 24vs. 111 ± 22, *p* = 0.000) and Tpe/QT ratio (0.29 ± 0.05 vs. 0.27 ± 0.05, *p* = 0.000) were increased. QTpc decreased during hypomagnesemia (334 ± 28 vs. 342 ± 21, *p* = 0.02). However, no significant differences were found in PR interval, QRS duration (85 ± 12 ms vs. 86 ± 12 ms, *p* = 0.122) and ST-T segments between the patients and their own controls.

**Conclusions:**

In patients with isolated hypomagnesemia, P wave duration, QTc, Tpec, and Tpe/QT ratio suggesting atrial depolarization and ventricular repolarization dispersion were significantly increased compared with normal magnesium levels in the same patients after restoration to normal levels.

## Introduction

Sudden cardiac death (SCD) has been widely studied because of its important public health implications (Ackerman et al., 2016); electrolyte disturbances have been thought to be related to its etiology. Magnesium is the second most abundant intra-cellular fluid cation, and is considered to be an important factor in facilitating influx of potassium ions into the myocyte, which relates to QT duration. Low serum magnesium is also regarded as a risk factor for ventricular tachycardia/fibrillation (VT/VF) and SCD (Adamopoulos et al., 2009; Del Gobbo et al., 2013; Kieboom et al., 2016). Moreover, administration of magnesium has been first line treatment for Torsades des Pointes (TdP; Neumar et al., 2010).

Studies focusing on isolated hypomagnesemia are limited. Early studies observed ST depression or T wave changes in magnesium deficient dogs (Seta et al., 1966). Other reports have suggested that low serum magnesium level is related to a higher incidence of various arrhythmias, including VT and VF and SCD (American Heart Association, 2000; Peacock et al., 2010). The possible mechanisms are thought to involve QT prolongation (Kieboom et al., 2016). However, these studies have, generally, not excluded hypokalemia, hypocalcemia and other electrolyte disturbances, or were based only on animal data. The electrocardiographic (ECG) characteristics of isolated hypomagnesemia remain not clearly defined. We, therefore, performed this study to investigate the specific changes in various ECG parameters among patients with isolated hypomagnesemia.

## Materials and Methods

### Study Design

The study population comprised patients admitted to the First Affiliated Hospital of Dalian Medical University between April 2011 and October 2017. The study sample collected subjects with documented isolated low serum magnesium and those same patients when magnesium levels were returned to the normal range as their own controls to offer comparison of ECG parameters between the two states. Seven-hundred-and-sixty-seven patients with intermittent low serum magnesium levels were selected from 270,997 patients from electronic medical records; The inclusion criteria were as follows:

(1) Patients with isolated hypomagnesemia (serum magnesium levels <0.65 mmol/L) without other electrolyte disturbance according to laboratory criteria in the First Affiliated Hospital of Dalian Medical University (serum calcium levels between 2.02 and 2.60 mmol/L, serum sodium levels between 137 and 147 mmol/L, serum potassium levels between 3.50 and 5.30 mmol/L, serum chlorine levels between 99 and 110 mmol/L and serum phosphate levels between 0.87 and 1.45 mmol/L).
(2) The patients in whom both normal and low magnesium levels were available, where the separate magnesium measurements were <3 months apart.
(3) ECGs were available contemporaneously with the blood samples. Two-hundred-and-fourteen of these 767 patients were shown to have isolated hypomagnesemia and 175 patients with intermittent low serum magnesium fulfilled all the above inclusion criteria. Additionally, those patients with diagnosis of bundle branch block, fascicular block, atrial flutter/fibrillation, atrioventricular block, VT were excluded.
(4) Patients taking QT-prolonging drugs were also excluded guided by crediblemeds.^1^
(5) Further, patients with end-stage heart failure results from various etiologies.

**FIGURE 1:**
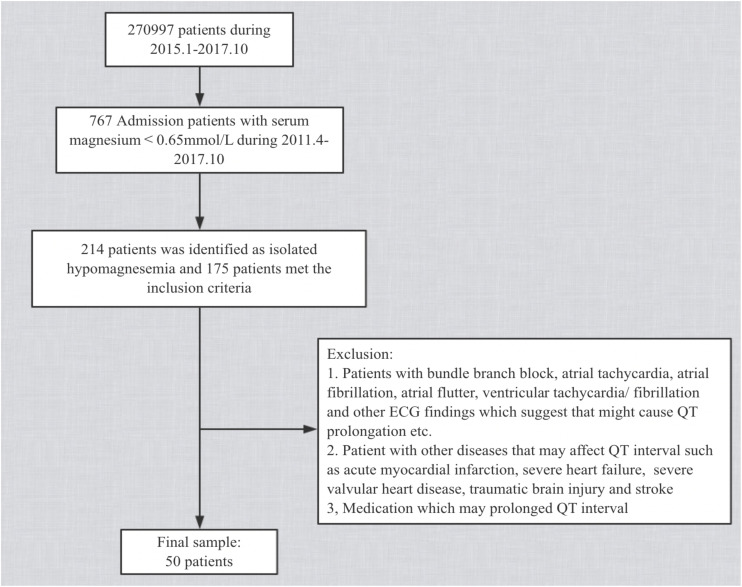
Flow chart of baseline screening of the study population.

After these exclusions, 50 patients were eligible for comparison with their own controls (Figure 1). Our study conforms to the Declaration of Helsinki and was approved by the Medical Ethics Committee of the First Affiliated Hospital of Dalian Medical University. Informed Patient consent was not required in our institution as this was a retrospective study according to local ethics rules.

### Assessment of Serum Magnesium

Fasting blood samples were drawn from an antecubital vein and sent immediately to the laboratory in the medical center. The timing of blood drawing and ECG recording was within 24 h. Magnesium was measured in serum by the clinical laboratory in the medical center using a Hitachi 7600-210 automatic analyzer (selective electrode method). Low serum magnesium levels were defined as <0.65 mmol/L. The laboratory coefficient of variation for Mg, based on split samples sent 1 week apart blindly to our laboratory, was 2.2–3.2%, rechecked on an annual basis.

### Measurements of ECGs

**FIGURE 2:**
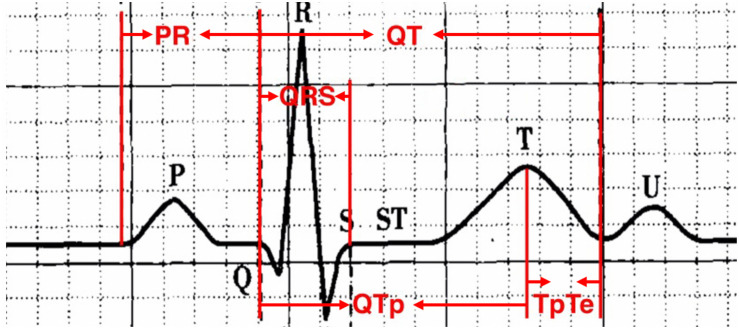
Definition of ECG parameters.

**FIGURE 3:**
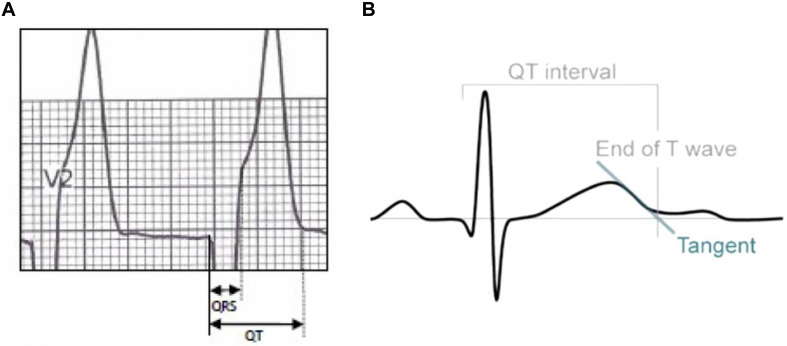
In normal T wave morphologies, the termination of the T wave was taken to be the point of maximal change in the slope as the T wave merges with the baseline **(A)**. If there was any difficulty in determining the end of the T wave, the end point is considered as the intersection between the tangent of steepest slope of the T wave and the isoelectric line **(B)**.

Standard 12-lead ECGs recorded by GE Healthcare, Chicago, IL, United States MAC5500 at a paper speed of 25 mm/s and voltage of 10 mm/mV. All ECG recordings were made with 40 Hz filtering which was carefully and correctly set in our GE machines. These devices offered some automated measurements but since we wished to record a number of measurements that were not provided, we opted to make all measurements manually. Definition of ECG parameters is shown in Figure 2. QT interval was manually measured as the beginning of the QRS complex to the endpoint of the T-wave. The terminal aspect of the T wave was identified as the point where the descending limb reaches the baseline. The methods of QT interval measurement have been shown in Figure 3 and well described in our previous publications (Wang et al., 2017; Yu et al., 2017; Vink et al., 2018). According to Bazett’s formula, QT-corrected (QTc) was defined as:

$$\text{QTc}={\text{QT}}_{\text{maximum}}/\sqrt{\mathrm{RR}\,\mathrm{interval}}$$

QT_maximum_ was measured from the earliest deflection of the QRS complex to the latest termination of T-wave among leads II, V2, and V5. Bazett’s formula was also used for correction of other parameters including corrected Tpe (Tpec), QTpc, and JT-corrected (JTc) interval. The other ECG parameters were measured as follows:

- T peak-to-end interval (Tpe) intervals were calculated in lead II, V2, V5;
- P wave duration was measured in lead II, V2 and V3;
- QT-peak (QTp) interval was measured from the beginning of the QRS complex to the peak of T-wave in lead II, V2, V5;
- the peak of the T wave was defined as the highest amplitude reached by the T-wave deflection;
- the longest Tpe interval from among the leads stated above was selected as Tpe_maximum_;
- the longest P wave duration_maximum_ and QTp_maximum_;
- JT interval was calculated by the difference between the QT_maximum_ and QRS duration.

Intervals were calculated as follows;

- Tpe interval = QT interval - QTp interval; Tpec = Tpe corrected;
- Tpe/QT ratio = Tpe_maximum_/QT_maximum_;
- Tpe dispersion = Tpe_maximum_ - Tpe_minmum_;
- QT dispersion = QT_maximum_ - QT_minmum_;
- P wave dispersion = P wave duration_maximum_ - P wave duration_minmum_;
- JT interval = QT_maximum_ - QRS duration;
- QTpc = QTp_*maximum*_/$\sqrt{\mathrm{RR}\,\mathrm{interval}}$;
- Tpec = Tpe/$\sqrt{\mathrm{RR}\,\mathrm{interval}}$.

According to the recommendation of the American Heart Association (AHA), T-wave amplitude was measured from the baseline to the peak of the T wave in lead II, V2, and V3 (Rautaharju et al., 2009). ST depression was defined as ST segments depressed >0.1mv in any lead. All ECG measurements were made by two cardiologists from among the authors who were blind to which group each patient belonged, and verified each other’s work. Measurements were performed on consecutive complexes on these leads (II, V2, and V5) whichever QT interval was the longest. We printed the ECG on A4 paper and indices were measured by calipers.

### Statistical Analyses

Based on the use of patients as their own controls described above, the electrolyte levels and ECG characteristics were compared between the two measurements, abnormal (serum magnesium <0.65 mmol/L) and normal (>0.65 to <0.8 mmol/L). Continuous variables were described by mean ± (standard deviation). The Student’s paired *t*-test was used to evaluate the difference for normally distributed continuous variables and Wilcoxon test for abnormally distributed variables. Kolmogorov–Smirnov test was used to test the distribution of continuous variables. Categorical variables were described in percentages and were compared using paired χ^2^ tests (McNemar’s test). We considered a two-sided *p*-value <0.05 to be statistically significant.

## Results

### Patient Characteristics

Of the 767 patients with low serum magnesium levels, 159 (20.7%) also had low serum potassium and 364 (47.5%) had both low serum potassium and calcium and, thus, were excluded. Table 1 shows the distribution of serum electrolytes stratified by serum magnesium level. No significant differences were found in other electrolytes in the included patients, neither in hypomagnesemia nor when normal.

**TABLE 1:** Serum electrolyte characteristics of patients stratified by serum magnesium.

	Hypomagnesemia group	Normal group	*P* value
Serum magnesium (mmol/L)	0.600.05	0.760.09	0.000
Serum potassium (mmol/L)	4.060.37	4.110.34	0.39
Serum calcium (mmol/L)	2.250.14	2.240.13	0.70
Serum sodium (mmol/L)	139.883.40	140.143.51	0.63
Serum chlorine (mmol/L)	104.084.13	103.584.24	0.46

*Study group <0.65 mmol/L, control group 0.65–0.8 mmol/L. Data are presented as mean ± standard deviation. *P* value derived from paired *t*-tests for normally distributed variables and Wilcoxon test for abnormally distributed variables.*

**FIGURE 4:**
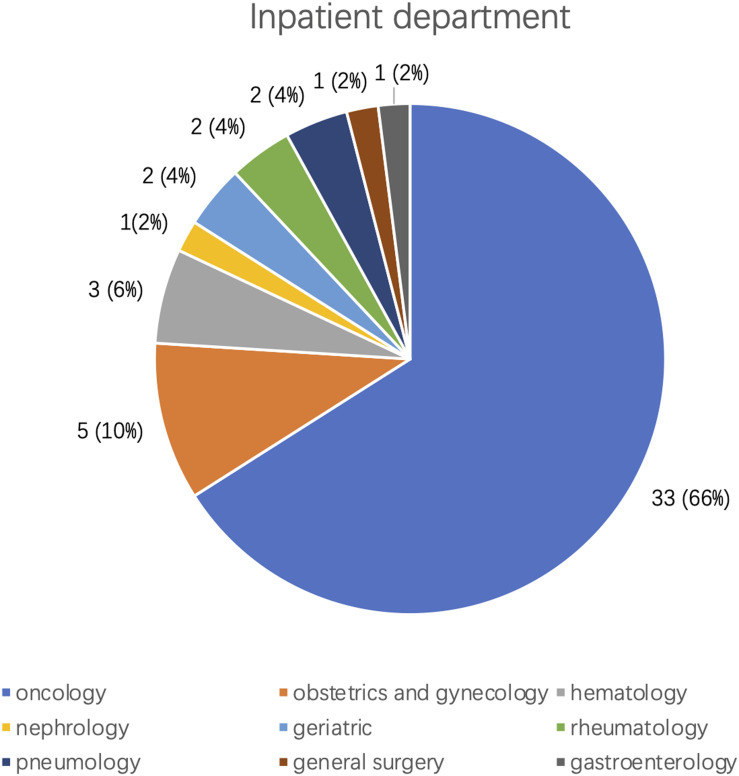
Inpatient departments distribution of the 50 patients with isolated hypomagnesemia.

We collected the clinical characteristics of the final studied sample, mean age 56.94 ± 13.63 years; males 50%. The average time between the two ECG recordings was 1.3 ± 0.8 (0.1–3.5) months. The common clinical diagnoses, including various cancers (38), hypertension (10), and diabetes (7), are shown in Table 2. Regarding the in-patient departments concerned (Figure 4), 66% patients (33/50) were from Oncology, the second most common department concerned was Obstetrics and Gynecology with 10% of patients, followed by Hematology (6%). Diagnoses including coronary artery disease/myocardial infarction/PCI history were not found.

**TABLE 2:** Baseline characteristics and clinical diagnosis of total sample.

	Study population
**Demographics**	
Mean age (Mean ± SD)	56.94 ± 13.63
Men (*N*, %)	25 (50%)
**Diagnosis (*N*)**	
Cancer	38
Hypertension	10
Diabetes	7
Anemia	3
Renal dysfunction	3
Pregnancy	4
Rheumatoid disease	4
Liver cirrhosis	3
COPD	2
Gout	2
Peptic ulcer	2
Arrhythmia	2
Stroke	1
Heart failure	1

*SD, standard deviation; COPD, chronic obstructive pulmonary disease.*

### Descriptions and Comparison of ECG Parameters

In the study group with isolated hypomagnesemia, we identified PR prolongation in 30 patients (60%). ST depression was found in 4 patients in both groups (8%). Amongst repolarization parameters, there were 24 patients (48%) with QTc prolongation, 26 (52%) patients with lower T wave amplitude in lead V2 compared with normal magnesium levels. Forty-six patients (92%) showed Tpec prolongation in lead V3 where was also the greatest increment (mean 16.9 ms). The ECG parameters of patients with low serum magnesium are presented in Table 3. Moreover, the inter-observer variability of QT measurement was calculated and provided in Table 4.

**TABLE 3:** ECG characteristics of patients with isolated hypomagnesemia.

	*N*	Percentage (%)
QRS interval shortening	30	60.0
PR interval prolongation	22	44.0
ST depression	4	8.0
QTc prolongation	24	48.0
**Tpec prolongation**		
Lead II	30	60.0
Lead V2	35	70.0
Lead V3	46	92.0
Lead V4	38	76.9
Lead V5	33	66.0
Lead V6	28	53.0
T wave amplitude peaking (lead II)	14	28.0
T wave amplitude peaking (lead V2)	26	52.0
T wave amplitude peaking (lead V3)	18	26.0

*QTc, QT corrected; Tpec, Tpeak to end corrected.*

**TABLE 4:** Inter-observer variability of QT interval measurements.

		Miss/cases	Observer 1	Observer 2	*P* value
Lead II	Study group (ms)	47/50	360 ± 38	363 ± 38	0.100
	Control group(ms)		363 ± 37	367 ± 36	0.062
Lead V2	Study group (ms)	50/50	365 ± 34	366 ± 35	0.496
	Control group (ms)		366 ± 37	369 ± 36	0.142
Lead V5	Study group (ms)	49/50	359 ± 34	362 ± 33	0.093
	Control group (ms)		365 ± 36	368 ± 36	0.171

We found that patients with isolated hypomagnesemia presented significantly higher Tpe/QT ratio (0.29 ± 0.05 vs. 0.27 ± 0.05, *p* = 0.000), longer QTc interval (439 ± 27vs. 433 ± 22, *p* = 0.01). and JTc interval (345 ± 29 vs. 338 ± 24, *p* = 0.05). Decreased QTpc interval (338 ± 28 vs. 342 ± 21, *p* = 0.02) and Tpec interval prolongation (122 ± 24 vs. 111 ± 22, *p* = 0.000) were also observed at the time of low magnesium. These patients with low magnesium also presented longer P wave duration in all measured leads. Notably, patients with low serum magnesium have shorter QRS duration (85 ± 12 vs. 87 ± 12, *p* = 0.122) but not significantly so. No significant differences were found in other ECG parameters between patients at time of isolated hypomagnesemia and with normal magnesium (Table 5).

**TABLE 5:** ECG parameter comparison between low serum magnesium and normal serum magnesium groups

		Hypomagnesemia group	Normal group	*P* value
	P wave dispersion	15 ± 8	16 ± 7	0.46
	**P wave duration (ms)**			
	Lead II	101 ± 21	93 ± 19	0.02
	Lead V2	94 ± 19	86 ± 19	0.005
	Lead V3	89 ± 19	82 ± 16	0.01
	PR interval (ms)	150 ± 21	149 ± 23	0.64
	QRS duration (ms)	85 ± 12	87 ± 12	0.122
	Heart rate (beats/min)	84.84 ± 16.50	82.33 ± 16.69	0.09
	ST depression	2(4.0)	2(4.0)	1.000
	QTc (ms)	439 ± 27	433 ± 22	0.01
	QTp_max (ms)	284 ± 38	298 ± 36	0.001
	QTpc (ms)	334 ± 28	342 ± 21	0.02
	Tpe_max (ms)	103 ± 20	96 ± 18	0.000
	Tpec_max (ms)	122 ± 24	111 ± 22	0.000
	JT_max (ms)	295 ± 36	296 ± 36	0.71
	JTc (ms)	345 ± 29	338 ± 24	0.05
	Tpe/QT	0.29 ± 0.05	0.27 ± 0.05	0.000
	Tpe dispersion	38 ± 16	34 ± 15	0.199
	QT dispersion	29 ± 19	27 ± 15	0.594
Lead II	QTpc (ms)	323 ± 27	332 ± 22	0.04
	Tpec	102 ± 25	89 ± 22	0.001
Lead V2	T ampltitude (mv)	0.22 ± 0.10	0.22 ± 0.11	0.97
	QTpc (ms)	313 ± 29	319 ± 25	0.15
	Tpec (ms)	117 ± 25	105 ± 23	0.000
Lead V5	QTpc (ms)	326 ± 31	332 ± 29	0.351
	Tpec (ms)	100 ± 21	95 ± 21	0.05

*Data are presented as mean ± standard deviation or number (%). *P* value derived from the paired *t*-test or Wilcoxon test (for continuous variables), and the paired χ^2^ test (for categorical variables) when appropriate.*

*QTc, QT corrected, Tpec, Tpeak to end corrected, QTp, QTpeak interval, QTpc, QTpeak corrected.*

**FIGURE 5:**
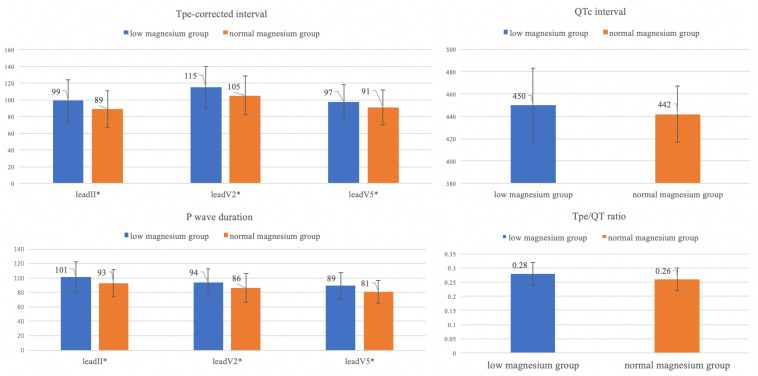
Differences of ECG parameters with significant changes (Tpec, QTc, P wave duration, Tpe/QT ratio). *P value < 0.05.

Difference of these ECG parameters with significant changes are shown in Figure 5.

## Discussion

Isolated hypomagnesemia has been relatively rare in our clinical practice. In this study, we analyzed the ECG parameters of patients with isolated hypomagnesemia, after excluding the concurrence of hypokalemia and/or hypocalcemia, and compared the findings with the same ECG parameters when normal serum magnesium levels were restored. We found that isolated hypomagnesemia was associated with longer P wave duration, QTc, Tpec interval and Tpe/QT ratio, indicating increased dispersion of ventricular repolarization.

### Role of Serum Magnesium *in vivo*

Magnesium, the second key intracellular cation in the human body, intracellular magnesium level varies between 5 and 20 mmol/L, depending on the different tissues, with the highest concentrations in skeletal and cardiac muscle (Kamonwan and Davenport, 2018). Consequently, serum magnesium level reflects only 1% of the body magnesium content and the clinical impact of magnesium deficiency on cardiac function may be underestimated (Jeroen et al., 2015). Magnesium exerts effects on cardiac function by regulating ion channels, plays a role in activation of Na+-K+ ATPase, which participates in transportation of K+ into cells and Na+ out of cells, and is a natural calcium antagonist. These functions could be adversely affected in magnesium deficiency, which, further, may result in hypokalemia and/or hypocalcemia and alteration of ECG characteristics. Previous studies have demonstrated that patients with hypertension and coronary heart disease are likely associated with more arrhythmias such as VT/VF and SCD (Peacock et al., 2010; Reffelmann et al., 2011). The Atherosclerosis Risk in Communities (ARIC) study also observed 45% SCD risk reduction in individuals with higher serum magnesium level (>0.87 mmol/L) compared with those with serum magnesium level <0.75 mmol/L (Peacock et al., 2010). Beneficial effects of magnesium in suppression of arrhythmia secondary to myocardial ischemia or quinidine have previously been reported. The main mechanism may be that magnesium can suppress the early afterdepolarization-induced triggered activity that may be responsible for initiating episodes of Torsade de Pointes and triggered activity suppression may be related to a “stabilizing” or surface charge effect (Davidenko et al., 1989). Elevated magnesium levels shift the threshold potential to less negative values and produce a membrane stabilizing effect which is similar but less than that of calcium (Castillo and Engbaek, 1954; Frankenhaeuser and Hodgkin, 1957). In addition, magnesium also acts to suppress triggered activity by inhibition of slow inward current (Isi) and consequently shorten phase 2 of the action potential, which is reported in canine Purkinje fibers (Sebeszta et al., 1981; Davidenko et al., 1989). Moreover, other studies have indicated that serum magnesium acts by blocking the increase in intracellular calcium during myocardial ischemia (Prielipp et al., 1995; Peacock et al., 2010). Consequently, low serum magnesium contributes to the increase in intracellular calcium, and results in calcium overload during acute ischemia and reperfusion, thus, playing an important role in arrhythmogenesis. Other possible pathways may contribute to the association between lower serum magnesium levels and dyslipidemia, metabolic syndrome, endothelial dysfunction, inflammation, atherosclerosis, and vascular calcification, all of which could be mechanisms to explain the above observations (Yang et al., 2016).

However, in these early studies almost all the baseline characteristics including serum potassium levels were significantly abnormal (*P* < 0.0001), which may confound the electrophysiological effect of depletion of serum magnesium. Zhang et al. (2012) reported that individuals with magnesium deficiency were found to have associated severe ischemic heart disease and fatal arrhythmias but there were no descriptions of ECG parameters including QTc in their regression model. Therefore, our results that reveal the relationship between QTc, Tpec, and Tpe/QT ratio suggesting increased ventricular repolarization dispersion may, therefore, verify the relationship between hypomagnesemia and SCD.

### P-QRS-T Complex

P wave duration was significantly prolonged with low magnesium levels. Increased P wave duration reflected slow or uncoordinated conduction in the atria. The mechanism is as yet unclear but may be a direct effect of low serum magnesium, or indirect by inhibition of K+ channels and result in a block of inward K+ current in phase 3 of the Action potential, considered as prolongation of effective refractory period. Consistent with our results in patients with hypomagnesemia, no difference was detected in PR interval in magnesium deficient beagles reported by Seta et al. (1966).

We identified a tendency to QRS shortening in 41 of 65 (63.1%) patients with low magnesium but it was not statistically significant. However, in an experimental study conducted in dogs (Syllm-Rapoport, 1962), Syllm-Rapoport (1962) found that dogs on a magnesium deficient diet had a shorter QRS duration in the resting state after several days but the observed abnormality could be attributed to diet, the resting state or other effects of the experiment. The Seta group’s study (Seta et al., 1966) detected widening of the QRS in the early stage of magnesium deficiency with peaked T waves and ST depression after 2 weeks. Thus, the most probable explanation for these differences is the fact that it is almost impossible to control all aspects of magnesium metabolism and maintain low serum magnesium levels for long durations in humans and in animals.

### QTc Interval and Tpe Interval

A recent study reported that hypomagnesemia was associated with QTc prolongation and SCD from the Rotterdam cohort (Kieboom et al., 2016). However, it must be noted that they did not report the baseline data on serum potassium and other electrolytes. Possible mechanism of QTc prolongation in hypomagnesemia patients is thought to be caused, at least in part, by the effect of hypokalemia or other electrolytes disturbances. Thus, our study may add some confirmation of the possible mechanism. Similarly, another earlier study showed a significant increase in QT interval in magnesium deficient dogs but QTc was not calculated (Syllm-Rapoport, 1962). The difference in results between these two studies (Syllm-Rapoport, 1962; Kieboom et al., 2016) could be due to changes in R–R interval. Subjects with hypomagnesemia in our study had slightly higher heart rates than those with normal magnesium levels potentially increasing QTc. Moreover, reduction in Mg^2+^ ions blocks inward flow of potassium which also potentially prolongs QTc. With respect to Tpec interval, generally, the apex of the T-wave represents epicardial repolarization while the end of the T-wave represents M-cell repolarization. The global dispersion of repolarization in different regions of the ventricular wall can be represented by Tpe and Tpe/QT ratio (Xia et al., 2005; Tse et al., 2018a). Tpe also reflects the length of the ventricular vulnerable period, increase in which has been thought to be a pro-arrhythmic factor for VT/VF in patients with prolonged QTc interval (Tse et al., 2017, 2018b). Recently, our team conducted a retrospective study comparing the outcomes of 293 patients with long QT syndrome (LQTS) compared with 542 patients without LQTS. LQTS patients, as expected, had higher mortality (Yu et al., 2017). Additionally, we found, in the present study, a significant increase in Tpec interval and Tpe/QT ratio among those with QTc prolongation, suggesting that serum magnesium could directly affect electrophysiologic characteristics via prolongation of Tpec. Low serum magnesium significantly shortened the QTpc interval in our study, which raises the possibility that QTc prolongation could be the result of increased Tpec. In terms of mechanism of these changes, it is possible that Tpec prolongation was observed in the early stage of serum magnesium deficiency and, at this early stage, the effect of hypomagnesemia on Na^+^-K^+^ ATPase is too small to have impact on serum potassium and QTc prolongation. Presently, the electrophysiologic action of low serum magnesium on cellular function is unclear but our results suggest Tpec interval prolongation may have importance in hypomagnesemic patients in clinical practice.

In this study, we draw a conclusion that atrial depolarization and ventricular repolarization dispersion were significantly increased in patients with isolated hypomagnesemia based on our results. However, we are unable to explain the reason why atrial and ventricular cardiomyocytes behave differently in electrolyte disturbances. There is currently insufficient knowledge on the influence of magnesium on the different phases of the cardiac action potential, and that it is also unknown whether there are different effects on different subtypes of cardiomyocytes or the specialized conduction system. Furthermore, we also think it should not be simply concluded that atrial and ventricular cardiomyocytes behave in opposite ways with this electrolyte disturbance, as atrial repolarization is not well reflected by the ECG, preventing us from being certain of the change in atrial repolarization. We anticipate that further study may illuminate this area in the future.

### Clinical Aspects

We identified 36 patients (72%) from Oncology and Hematology departments, the main diagnosis including lung cancer (25), leukemia (2), and lymphoma (3) that accounted for almost 80% of the total. We investigated the patients’ medications and found that some chemotherapeutic drugs increase renal magnesium loss by inhibition of reabsorption. Use of cisplatin has been found to be associated with renal magnesium loss, by protein-binding adversely affecting distal tubular reabsorption of magnesium in up to 90% of cancer patients (Goren, 2003; Taguchi et al., 2005) and 19 of our total of 50 patients received intravenous cisplatin. Two patients received ifosfamide or amphotericin B, both of which may cause distal renal tubular damage, and thereby reduce serum magnesium (Klastersky, 2003; Goldman and Koren, 2004). Furthermore, leukemic cells could affect renal tubular function increasing magnesium excretion (Miltiadous et al., 2008). Five patients (10%) from the Obstetrics and Gynecology department were included in our study where magnesium metabolism may be critical; reduction of serum magnesium can enhance uterine sensitivity, thus, helping to initiate delivery in late pregnancy. Moreover, serum magnesium deficiency plays a key role in eclampsia.

### Strengths and Limitations

There are limitations of our study to be acknowledged; the study has been performed in a single center, we only have a single measurement of serum magnesium without serial measurements. This methodological limitation also pertains to other studies in this field (Kieboom et al., 2016; Wannamethee et al., 2018). Further, we are unable to be certain of the temporal association between hypomagnesemia and SCD because we were unable to obtain the clinical outcome of all patients. Indeed, we may have failed to obtain all subtle changes during the fluctuation of magnesium in these patients. Preclinical studies focus on the wedge preparations, single myocardial cells may reveal more details of pathological changes in the future.

The strengths of our study are inclusion of only isolated hypomagnesemia excluding other electrolyte disturbances, patients acting as their own controls, and the availability of much of the clinical picture. Since isolated hypomagnesemia is relatively rare, monitoring of serum electrolytes including magnesium is of great value in prompting the maintenance of electrolyte balance which may reduce the risk of life-threating arrhythmia and SCD. In clinical practice, hypomagnesemia which is also likely to lead to serious clinical conditions such as SCD is not scrutinized by physicians as much as hypokalemia. More detailed descriptions of ECG characteristics of hypomagnesemia may trigger greater attention to this abnormality. Furthermore, prolongation of Tpec and Tpe/QT ratio indicating increased dispersion of ventricular repolarization can bring more understanding of the relationship between hypomagnesemia and SCD. Further clinical trials are needed in order to determine the clinical benefit and ECG characteristics of magnesium supplementation.

## Conclusion

In patients with isolated hypomagnesemia, P wave duration, QTc, Tpec, and Tpe/QT ratio suggesting atrial depolarization and ventricular repolarization dispersion were significantly increased compared with normal magnesium levels in the same patients after restoration to normal levels.

## Data Availability Statement

The raw data supporting the conclusions of this article will be made available by the authors, without undue reservation.

## Author Contributions

YX designed the study in which RS also played a part. YY and XY wrote the manuscript. CC, ST, SH, RT, YW, and RZ conducted the patient selection and the ECG measurements. YY maintained the database and conducted data analysis. XY and YD provided the electronic medical record database and reviewed the flow of study. RS, LG, and YX conducted the quality assurance and reviewed and edited the manuscript. All authors contributed to the article and approved the submitted version.

## Conflict of Interest

The authors declare that the research was conducted in the absence of any commercial or financial relationships that could be construed as a potential conflict of interest.

## Footnotes

1 https://www.crediblemeds.org/new-drug-list/
